# Induction of Neural Crest Stem Cells From Bardet–Biedl Syndrome Patient Derived hiPSCs

**DOI:** 10.3389/fnmol.2019.00139

**Published:** 2019-06-21

**Authors:** William B. Barrell, John N. Griffin, Jessica-Lily Harvey, HipSci Consortium, Richard Durbin, Davide Danovi, Philip Beales, Agamemnon E. Grigoriadis, Karen J. Liu

**Affiliations:** ^1^Centre for Craniofacial and Regenerative Biology, King’s College London, London, United Kingdom; ^2^School of Psychology and Neuroscience, University of St Andrews, St Andrews, United Kingdom; ^3^Centre for Stem Cells & Regenerative Medicine, King’s College London, London, United Kingdom; ^4^Genetics and Genomic Medicine Programme, UCL Great Ormond Street Institute of Child Health, London, United Kingdom

**Keywords:** neural crest, human induced pluripotent stem cells, hiPSCs, Bardet–Biedl Syndrome, BBS

## Abstract

Neural crest cells arise in the embryo from the neural plate border and migrate throughout the body, giving rise to many different tissue types such as bones and cartilage of the face, smooth muscles, neurons, and melanocytes. While studied extensively in animal models, neural crest development and disease have been poorly described in humans due to the challenges in accessing embryonic tissues. In recent years, patient-derived human induced pluripotent stem cells (hiPSCs) have become easier to generate, and several streamlined protocols have enabled robust differentiation of hiPSCs to the neural crest lineage. Thus, a unique opportunity is offered for modeling neurocristopathies using patient specific stem cell lines. In this work, we make use of hiPSCs derived from patients affected by the Bardet–Biedl Syndrome (BBS) ciliopathy. BBS patients often exhibit subclinical craniofacial dysmorphisms that are likely to be associated with the neural crest-derived facial skeleton. We focus on hiPSCs carrying variants in the *BBS10* gene, which encodes a protein forming part of a chaperonin-like complex associated with the cilium. Here, we establish a pipeline for profiling hiPSCs during differentiation toward the neural crest stem cell fate. This can be used to characterize the differentiation properties of the neural crest-like cells. Two different *BBS10* mutant lines showed a reduction in expression of the characteristic neural crest gene expression profile. Further analysis of both *BBS10* mutant lines highlighted the inability of these mutant lines to differentiate toward a neural crest fate, which was also characterized by a decreased WNT and BMP response. Altogether, our study suggests a requirement for wild-type BBS10 in human neural crest development. In the long term, approaches such as the one we describe will allow direct comparison of disease-specific cell lines. This will provide valuable insights into the relationships between genetic background and heterogeneity in cellular models. The possibility of integrating laboratory data with clinical phenotypes will move us toward precision medicine approaches.

## Introduction

Neural crest cells are a highly migratory, multipotent stem cell population that contributes to a broad range of tissues, including craniofacial bone and cartilage, peripheral neurons, glia, pigment, and other cells during embryonic development (Trainor, 2014). Elucidating the cellular and molecular mechanisms of lineage specification and migration of neural crest stem cells is essential for understanding the pathogenesis of neurocristopathies, a family of diseases caused by anomalies in the migration or cell behavior of neural crest cells. Ciliopathies are a class of genetic disorders characterized by mutation of proteins affecting the structure or function of the cilium (Eggenschwiler and Anderson, 2007; Ishikawa and Marshall, 2011). The cilium is a microtubule-based cellular organelle crucial for cell signaling, mechanotransduction and fluid flow, such as in airways and brain ventricles. Loss of the cilium has been linked to craniofacial anomalies such as micrognathia and facial clefting (Cortés et al., 2015; Adel Al-Lami et al., 2016). Cranial ciliopathies are caused by mutations in ciliary genes that may result from altered neural crest cell development (Cortés et al., 2015). How neural crest cell migration (see Tobin et al., 2008; Schock et al., 2015) is affected by cilia defects remains to be fully explored. Bardet–Biedl Syndrome (BBS) is a prototypic ciliopathy that can affect neural crest-derived tissues.

**FIGURE 1:**
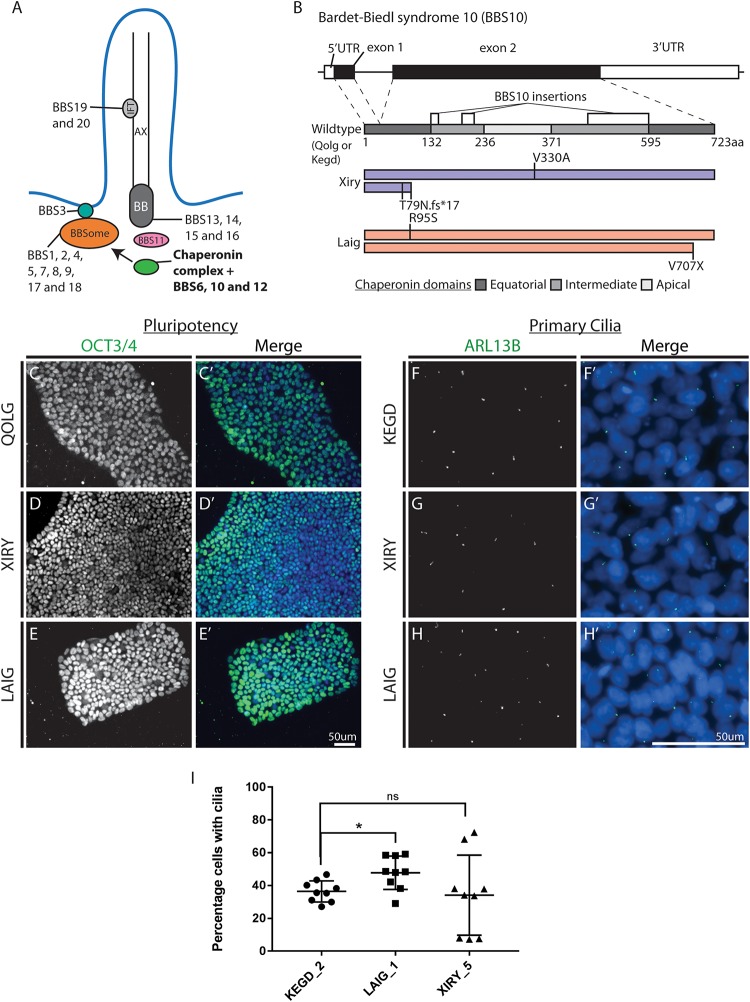
BBS10 variant hiPSCs are pluripotent and can form cilia. **(A)** Schematic representation of the primary cilium. AX, axoneme; BB, basal body; IFT, intraflagellar transport. BBS1-20 proteins are depicted in their associated complex (BBSome and chaperonin) or by localization to other structures (scheme adapted from Suspitsin and Imyanitov, 2016). **(B)** Schematics showing *BBS10* transcript and protein structure (723AA). Regions exhibiting homology with chaperonin domains are shown in the wildtype protein (scheme adapted from Álvarez-Satta et al., 2017). Variants from two *BBS10* mutant lines XIRY (blue) and LAIG (red) are mapped onto protein domains with comparison to control (QOLG or KEGD). **(C–E)** Cells were immunostained for the pluripotency marker OCT3/4. Control (QOLG) cells and *BBS10* mutants (XIRY and LAIG) all had positive staining in the nucleus. Merge with DNA dye Hoechst **(C’–E’)**. All cells exhibit staining although there is some variability with less intense staining being seen in the central regions of the colonies. **(F–H)** Staining for the ciliary axoneme marker, ARL13B was performed. Control (KEGD) cells show positive staining for ARL13B **(F)**. XIRY cells and LAIG cells **(H)** both express cilia. Merge with DNA dye Hoechst **(F’–H’)**. Cilia frequency was quantitated by manual counting **(I)**. LAIG cells had a moderate increase in percentage of cells with cilia compared to control cells. XIRY cells showed no significant difference although were more variable. *P*-values were determined using unpaired Student’s *t*-tests (^*^P ≤ 0.05). Scale bars **(E’,H’)** = 50 μm.

BBS proteins can be categorized by their location and function within the primary cilia, with a subset of BBS proteins forming the BBSome (Figure 1A). The BBSome is an adaptor complex that is crucial for cilia function (Jin and Nachury, 2009; Suspitsin and Imyanitov, 2016). Clinical findings indicate that patients carrying *BBS* mutations often present with subclinical craniofacial changes including speech and oral phenotypes, mid-facial flattening and some mild retrognathia (Tobin et al., 2008; Panny et al., 2017). In addition, *Bbs6* mutant mouse skulls showed a reduction in the size of the pre-maxillary and maxillary regions (Tobin et al., 2008). Similarly, zebrafish knockdowns of *bbs4*, *bbs6*, and *bbs8* had shortened facial cartilages and mandibles correlating with a reduction in neural crest cell migration (Tobin et al., 2008).

We focused on *BBS10* due to the high prevalence of *BBS10* mutations in humans, which comprises approximately 20% of the BBS population (Stoetzel et al., 2006; Forsythe and Beales, 2013). BBS10, along with BBS6 and 12, is part of a chaperonin-like complex which mediates the assembly of the BBSome (Stoetzel et al., 2006, 2007; Billingsley et al., 2010; Seo et al., 2010; Zhang et al., 2012). Phenotypes associated with *BBS6, 10* and *12* are thought to be more severe than other commonly mutated BBS genes like *BBS1* (Castro-Sánchez et al., 2015). Furthermore, *bbs10* morphant zebrafish larvae exhibit shortened body axis and poor somitic definition among other more variable phenotypes, while a sub-phenotypic dose of *bbs10* morpholino oligonucleotides (MO) exacerbates the phenotypes observed in other *bbs* morphants (Stoetzel et al., 2006). In alignment with the human disease, *Bbs10* knockout mice are viable but exhibit obesity, retinal degeneration and cystic kidney phenotypes (Cognard et al., 2015).

Together, these studies in fish and mouse suggest that BBS10 is necessary for normal cilia function, and when mutated, causes a range of BBS phenotypes. The potential neural crest defects observed in the knockdowns also fit generally with observations linking ciliopathic mutations and craniofacial malformations in humans. It is unclear what specific alterations in neural crest cell (NCC) functions (e.g., differentiation, migration) are caused by mutations in BBS10. To define the pathogenic mechanisms of these mutations, we sought to model NCC induction in BBS10 mutant iPSCs.

Human induced pluripotent stem cells (hiPSC) are a powerful tool for the study of human developmental disorders as they can be used to model genotype-specific molecular and cellular phenotypes during differentiation. Over the last decade, several protocols have been developed for neural crest cell (NCC) induction from hiPSCs, using defined factors at specific time points to generate “purer” cell populations which can be used to study induction, migration and differentiation (Chambers et al., 2013; Menendez et al., 2013; Leung et al., 2016; reviewed in Zhu et al., 2016). The Human Induced Pluripotent Stem Cell Initiative (HipSci^1^) has generated and characterized a panel of hundreds of hiPSCs including from a significant cohort of BBS patients hosting a diverse spectrum of mutations in genes involved in BBS. Here, as a proof of concept, we use the recently described (Leung et al., 2016) protocol to obtain efficient neural crest induction and compare HipSci cell lines from healthy volunteers with two cell lines obtained from BBS patients carrying *BBS10* mutations (Table 1). We show that both mutant cell lines have altered neural crest induction and differentiation, demonstrating the feasibility of using hiPSCs from ciliopathies to explore processes affecting neural crest induction. This approach will help us to define the human phenotypes and dissect the role of cilia during neural crest development.

**TABLE 1:** Cell lines used in this study.

**Donor**	**Cell line**	***BBS10* variant 1**	**BBS10 variant 2**	**Age**	**Sex**	**Ethnicity**
QOLG	Qolg_1	Wildtype	Wildtype	35–39	Male	White, British
KEGD	Kegd_2	Wildtype	Wildtype	40–44	Male	White, British
XIRY	Xiry_5	c.989T>C/p.Val330Ala	c.235dup/p.Thr79Asnfs^*^17	35–39	Male	White, British
LAIG	Laig_1	c.285A>T/p.Arg95Ser	c.2119_2120del/p.Val707^*^	25–29	Female	White, European

## Materials and Methods

### Human Induced Pluripotent Cell Lines (hiPSC)

Control hiPSCs and BBS patient-derived hiPSC lines harboring mutations in *BBS10*, part of the ciliary chaperonin complex (Figure 1A) were obtained from the HipSci consortium (see Table 1). Ethical approval was obtained either from NRES Committee East of England – Cambridge Central, Study title: Generation of Induced Pluripotent Stem (iPS) Cells and Rare Diseases. REC Ref: 15/EE/0049, or from NRES Committee London-Bloomsbury, Study title: Molecular Genetics of Human Birth Defects – mapping and gene identification. REC Ref: 08/H0713/82. hiPSCs were maintained in Essential 8 media (Thermo Fisher Scientific, United States) on an extracellular matrix replacement, Matrigel (Corning). XIRY was initially obtained cultured on Matrigel whereas QOLG and LAIG, acquired on vitronectin, were cultured on Matrigel for several passages to ensure that all cell lines were comparable. Cells were typically passaged twice weekly using 0.5 mM EDTA, dissociated with gentle pipetting and analyzed between passages 30–60.

### Neural Crest Induction

**FIGURE 2:**
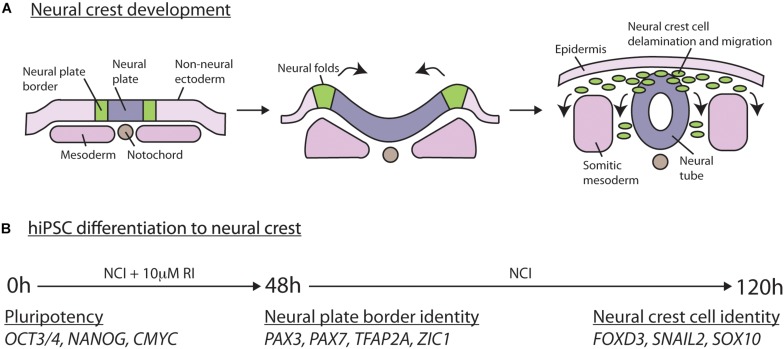
Neural crest induction *in vivo* and in human induced pluripotent stem cells. **(A)** *In vivo* neural crest induction occurs at the border (green) between the neural plate (blue) and the non-neural ectoderm (light pink). As neurulation progresses the neural folds begin to approximate and neural crest delaminate from the epithelium and begin migration. **(B)** *In vitro* neural crest induction in hiPSCs following the protocol from Leung et al., 2016. hiPSCs are plated on Matrigel and treated for 48 h in neural crest induction (NCI) media plus Rock inhibitor (RI). This is followed by incubation in neural crest induction media without RI until 120 h of culture. At the start, pluripotency markers (*OCT3/4, NANOG*, and *CMYC*) are expressed. By 2 days of culture, neural border markers are expressed (*PAX3, PAX7, TFAP2A, ZIC1*). By 5 days of culture, cells express markers of neural crest identity (*FOXD3, SNAIL2, SOX10*).

Neural crest induction was carried out as per (Leung et al., 2016), which is designed to mimic normal neural crest development (Figure 2). TrypLE Express (Thermo Fisher Scientific, United States) was used to detach hiPSCs into a single cell suspension. Next, 200,000 hiPSCs were plated in each well of a 6-well plate coated with Matrigel. At the start of the induction protocol (time = 0 h), wells were incubated with neural crest induction medium + 10 μM Y-27632 (Cell Guidance Systems) for the first 48 h (48 h) of culture, then in neural crest induction media alone for the indicated time. Samples were then fixed in 4% PFA in PBS for antibody staining or lysed in Trizol (Sigma) for RNA extraction. Neural crest induction media is as per (Leung et al., 2016): 50:50 DMEM:F12 (Sigma, United States), 2% B27 supplement (Life Technologies, United States), 1X L-glutamine (Invitrogen), 0.5% BSA (Sigma), 3 μM CHIR99021 (Sigma).

### Immunofluorescent Antibody Staining

After fixation, cells were washed three times in 1X PBS and then permeabilised by incubation with 0.5% TritonX-100 (Sigma) in 1X PBS for 5 min at room temperature. Next, cells were blocked for 1 h at room temperature [in 3% BSA (Sigma) in 1X PBS, 0.01% Tween 20 (Sigma)]. Primary antibodies (see Table 2) were incubated overnight at 4°C in blocking solution. Three 15 min washes in PBS with 1% BSA, 0.01% Tween 20 were carried out. Fluorescent secondary antibodies were diluted in blocking solution and plates were incubated at room temperature for 1–2 h. Next, three 15 min washes in PBS with 1% BSA, 0.01% Tween 20 were carried out. Hoechst 33342 (Sigma), diluted at 1:1000 from a 20 mg/ml stock, was added to the first wash as a nuclear stain.

**TABLE 2:** Antibodies used in this study.

**Antibody**	**Targets:**	**Concentration**	**Supplier (catalog number)**
ARL13B	Ciliary axoneme	1:500	Proteintech (17711-1-AP)
SOX10	Migratory neural crest	1:500	Santa Cruz (sc-365692)
OCT3/4	Pluripotency	1:200	Santa Cruz (sc-5279)
P75NTR	Neurotrophin receptor/neural crest	1:600	Millipore (07-476)
Anti-mouse 488	Secondary antibody	1:500	Invitrogen (A11001)
Anti-rabbit 568	Secondary antibody	1:500	Invitrogen (A11011)
Anti-rabbit 488	Secondary antibody	1:500	Invitrogen (A11008)

### Microscopy and Imaging

Cells were routinely viewed and imaged by phase-contrast microscopy. Fluorescence imaging of immunostained samples was carried out using an inverted epifluorescence microscope (Zeiss or Olympus) with a plate stage adaptor. All image processing was carried out in Image J. Figures were made using Adobe Illustrator CS5.5.

Cilia quantification was manually carried out using the cell counter plug-in of ImageJ. For these quantifications the control line KEGD was used due to lack of QOLG line availability. In addition, these cells were cultured on vitronectin rather than Matrigel (Corning). The percentage of ciliated cells was calculated from total nuclei and cilia counts manually obtained from representative images (total number of control cells counted: KEGD = 2631, mutant cells: LAIG = 2293 and XIRY = 1742). Nuclei and cilia of cells touching the field edge were assessed from contrast adjustment of the ARL13B channel and excluded.

### Quantitative Reverse Transcription Polymerase Chain Reaction (qRT-PCR)

RNA was extracted from hiPSCs, after 48 h of induction or 120 h of induction, using Trizol (Sigma) as per manufacturer’s instructions. RNA was then DNAse I (Promega, United States) treated. 500 ng to 1 μg of DNAse treated RNA was primed with random hexamers (Promega) and reverse transcription was carried out according to manufacturer’s protocol (M-MLV Reverse Transcriptase, Promega, M1701. All qRT-PCRs were performed using Bioline 2X Sensimix (QT605-05) using a Roche Lightcycler 480. Quantification of each PCR reaction was carried out using absolute standards, and normalized to β-*actin* levels. See Table 3 for primers used in this study. Statistical significance for all RT-qPCR data was analyzed using un-paired, Student’s *t-*tests.

**TABLE 3:** RT-qPCR primers used in this study.

**Gene**	**Forward primer**	**Reverse primer**
*β-ACTIN*	AGCCTCGCCTTTGCCG	CTCGTCGCCCACATAGGAAT
*CMYC*	TTTCTGAAGAGGACTTGTTGCGGAAACGAC	TCAGCCAAGGTTGTGAGGTTGCATTTGATC
*NANOG*	TCTCTCCTCTTCCTTCCTCCATG	CTGTTTGTAGCTGAGGTTCAGGATG
*OCT3/4*	GACAGGGGGAGGGGAGGAGCTAGG	CTTCCCTCCAACCAGTTGCCCCAAAC
*BRY*	TATGAGCCTCGAATCCACATAGT	CCTCGTTCTGATAAGCAGTCAC
*AXIN2*	AGTCAGCAGAGGGACAGGAA	AGCTCTGAGCCTTCAGCATC
*PTC1*	CCCCTGTACGAAGTGGACACTCTC	AAGGAAGATCACCACTACCTTGGCT
*SIX1*	TCAGCTCCAAGACTCTCTGC	ACAAGCTGCAAAAATGTTCC
*SOX2*	TCAAGCGGCCCATGAATGCC	AGCCGCTTAGCCTCGTCGAT
*TFAP2A*	GATCCTCGCAGGGACTACAG	TACCCGGGTCTTCTACATGC
*ZIC1*	GTCCTACACGCATCCCAGTT	GCGATAAGGAGCTTGTGGTC
*MSX1*	CTGCACCCTCCGCAAACACA	AGGCTGAGCGAGCTGGAGAA
*PAX3*	AATTACTCAAGGACGCGGTC	TTCTTCTCGCTTTCCTCTGC
*PAX7*	TGACAGCTTCATGAATCCGG	GATGGAGAAGTCAGCCTGTG
*FOXD3*	GCATCTGCGAGTTCATCAGC	CGTTGAGTGAGAGGTTGTGG
*SOX10*	CTCTGGAGGCTGCTGAA	TGGGCTGGTACTTGTAGTC
*SNAI2*	CAGACCCTGGTTGCTTCAAG	GAGCCCTCAGATTTGACCTG

## Results

### Comparable Culture Conditions for Pluripotent Stem Cells

We selected several lines from the HipSci cell bank: a control line, KEGD (cilia quantitation) and QOLG (used for the majority of our assays) and two *BBS10* mutant lines, XIRY and LAIG. Both mutant lines carry compound heterozygous variants (Figure 1B and Table 1). The XIRY line carries two alleles: a non-synonymous change (V330A) on one allele, and a duplication on the other allele that leads to a frameshift and an early stop (T79Nfs^*^17), likely to result in a loss-of-function variant (Figure 1B). LAIG carries an allele with a synonymous mutation (R95S) and an allele with a 2-base pair deletion leading to a truncated protein lacking the final 16 amino acids (V707^*^) (Figure 1B). Based on these genotypes, we expect that the XIRY variants will have a more significant impact on protein function. Matrigel was used to wean lines for comparable results and all cell lines exhibited an embryonic stem (ES) cell-like state, forming tight colonies of packed cells (Figures 1C–E). Cultures were discarded if delaminating, if differentiated cells were present on greater than 25% of the colonies, or if large regions of non-colony bound cells were present. We confirmed that our culture conditions were suitable for maintaining pluripotency in all three hiPSC lines. Immunostaining for OCT3/4, one of the defining features of pluripotency, was carried out on all three lines after adaptation to Matrigel. All lines exhibited positive nuclear staining for OCT3/4 confirming the pluripotent nature of these cells, and no significant differences were observed in *BBS10* mutants compared to controls (Figures 1C–E).

### Reduced Primary Cilia Expression in BBS10 Mutant hiPSCs

Normally, a proportion of cells within hiPSC colonies express primary cilia (Nathwani et al., 2014). To confirm that *BBS10* mutant cells are still able to generate cilia, we performed immunostaining on the hiPSCs with ARL13B, a marker of the ciliary axoneme. We found that both control lines expressed an abundance of cilia-specific ARL13B staining (KEGD, shown in Figure 1F; QOLG, data not shown). Both *BBS10* mutant lines also had cells with positive staining (Figures 1G,H). Both mutant lines expressed cilia; interestingly, quantification indicated that LAIG and not XIRY had a slight but statistically significant increase in the number of cilia numbers compared to control (Figures 1F–I). Anecdotally, the percentage of cells with cilia in XIRY was very variable (Figures 1G,I). These data suggest that *BBS10* is not strictly required for ciliogenesis, at least in the pluripotent state.

### Differentiation of hiPSCs to Neural Crest Stem Cells

**FIGURE 3:**
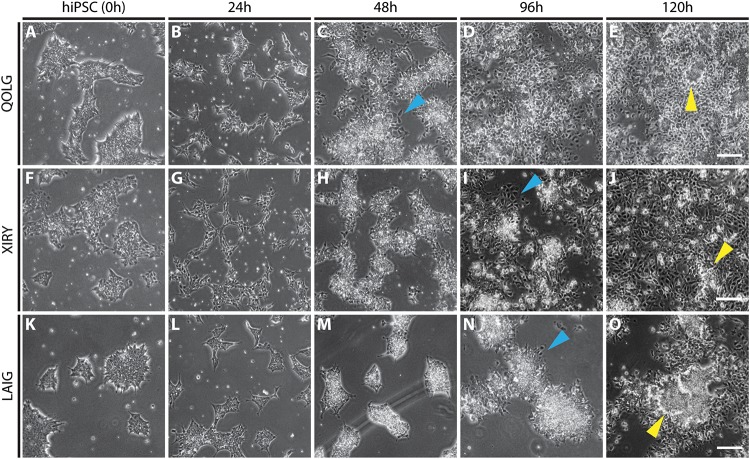
Neural crest induction is delayed in BBS10 mutant cells. Phase contrast images of taken during neural crest induction at specified time points. Control QOLG **(A–E)**, mutant XIRY **(F–J),** and LAIG **(K–O)** cells. Discrete iPSC-like colonies can be seen at 24 h in all rows **(B,G,L)**. In control cultures, cells are seen delaminating from colonies at 48 h (blue arrowhead, **C)**; whereas mutant cultures start showing delamination at 96 h (blue arrowheads, **I,N)**. By 120 h there are still some dense, colony-like regions present in all cultures (yellow arrowheads, **E,J,O**). Scale bars = 50 μm.

To confirm that both normal and BBS10 mutant hiPSCs could be differentiated toward the neural crest lineage, we used a 5-day induction protocol where activation of the WNT pathway [via inhibition of glycogen synthase kinase-3 (GSK-3)] induces neural border genes and neural crest markers mimicking normal neural crest development (Figure 2; Leung et al., 2016). Cells were monitored daily and unattached cells were washed away during media changes, leaving behind adherent colonie in control (Figures 3A–E) and mutant cells (Figures 3F–J,K–O). At initial plating, control and mutant cells formed small colonies with protrusions (Figures 3A,F,K). By 24 h of culture, cells are still present as small iPS-like colonies (Figures 3B,G,L). By 48 h of culture, a subset of cells in the control QOLG colonies have begun delaminating (Figure 3C, blue arrowhead). This was rarely seen in *BBS10* mutant colonies at 48 h (Figures 3H,M). In contrast, mutant cell colonies showed delamination at 96 h of culture (Figures 3I,N, blue arrowheads). These results suggest a delay in neural crest induction in BBS10 mutants.

**FIGURE 4:**
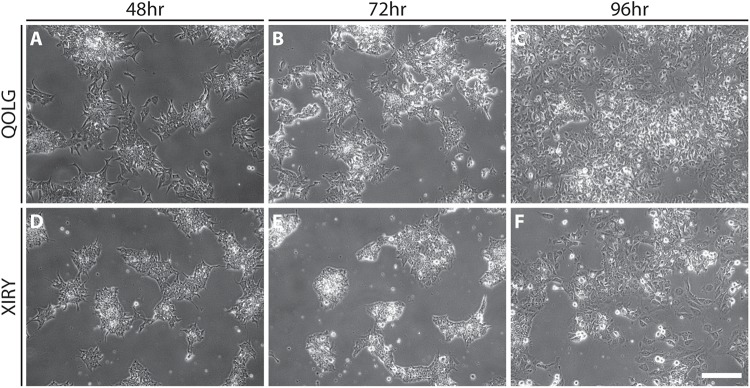
Delaminated neural crest cells have a mesenchymal morphology. Phase contrast images of differentiating hiPSCs cultures taken during neural crest induction at specified time points. Control QOLG **(A–C)**, mutant XIRY **(D–F)**. Note that control QOLG cells at 48 h **(A)** exhibit delaminated cells with mesenchymal morphology, in contrast to XIRY cells which do not show cell delamination until 96 h **(F)**. Scale bars = 200 μm.

Furthermore, despite being initially plated at the same density, fewer cells were observed in the mutant cultures over the first 72 h of culture. By day 5, most areas of cultures had reached confluency; however, the control was visibly denser (Figure 3E). At 120 h dense areas (yellow arrowheads, Figures 3E,J,O) were present, and these resembled the morphology of colony-like arrangements seen throughout the cultures. Upon close inspection of the delaminating stage of QOLG and XIRY cells (48–96 h, Figure 4), it was noted that XIRY cells showed fewer protrusions from the colonies of cells at 48 and 72 h timepoints (Figures 4D,E). At 96 h mutant cells are seen delaminated from cell colonies (Figure 4F). These data indicate that some neural crest induction is taking place, with cells delaminating from control colonies earlier than mutants.

### Decreased Expression of Neural Crest Markers in Mutant Cells

**FIGURE 5:**
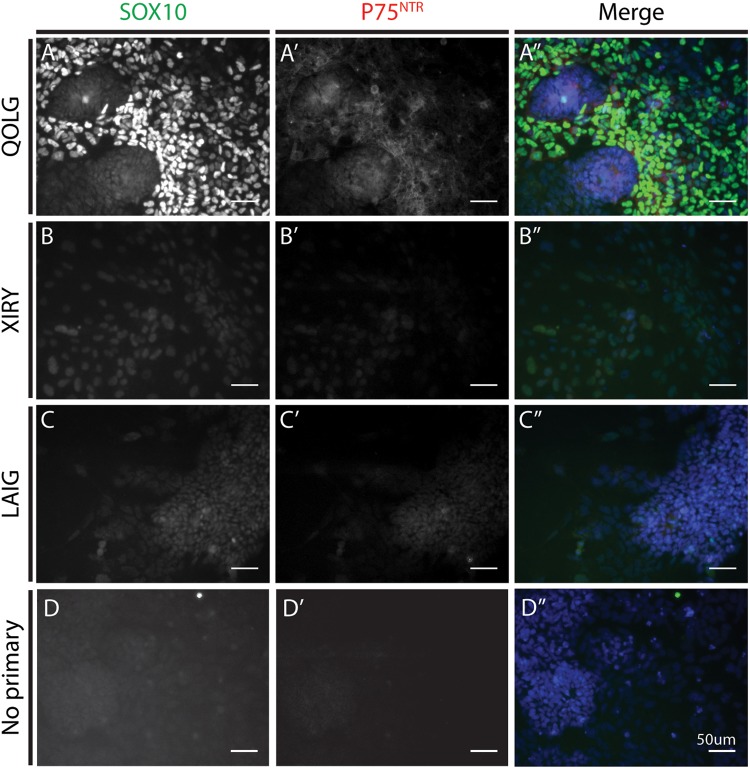
*BBS10* mutant cells express lower levels of SOX10 and P75NTR after neural crest induction. Immunostaining for the neural crest markers SOX10 **(A–C)** and P75NTR **(A’–C’)**. Control QOLG **(A)** cells had high levels of SOX10 and P75NTR. *BBS10* mutant cells [XIRY **(B)** and LAIG **(C)**] had significantly lower levels of both markers. Merge with DNA dye Hoechst **(A”–C”)**. No positive staining was observed in the no primary control for SOX10 or P75NTR **(D–D”)**. Scale bars = 50 μm.

During embryogenesis, the transcription factor SOX10 and the p75 neurotrophin receptor (p75^NTR^) are expressed in migratory neural crest cells. In order to determine the identity of the induced cells, we carried out immunostaining for these markers. By 5 days of differentiation a majority of cells showed some level of SOX10 expression (Figure 5). The QOLG control line showed much higher levels of SOX10 and p75^NTR^ expression compared to the mutant lines (Figures 5A–C). Consistent with SOX10 association with migratory neural crest cells, cells that most strongly expressed SOX10 were delaminated cells at the edge of colonies (Figure 5A). We also found cells co-expressing p75^NTR^ and SOX10 around the periphery of control colonies (Figures 5A–A”). In contrast, both mutant cultures had markedly diminished expression of SOX10 and almost no evidence of p75NTR expression (Figures 5B–C”), although levels of SOX10 were still above background (compare Figures 5D,B–C).

### Gene Expression Analysis During Induction of the Neural Crest Lineage

As noted above, the neural crest protocol is designed to first induce a neural border state, which is necessary for subsequent neural crest induction. In order to determine the steps at which neural crest induction might be perturbed, we examined gene expression profiles over the 120 h protocol. We harvested mRNA for quantitative RT-PCR analysis at 0 h, where cells should be self-renewing hiPSCs, at 48 h induction, at the onset of neural border formation, and at 120 h, when cells should be expressing neural crest markers.

**FIGURE 6:**
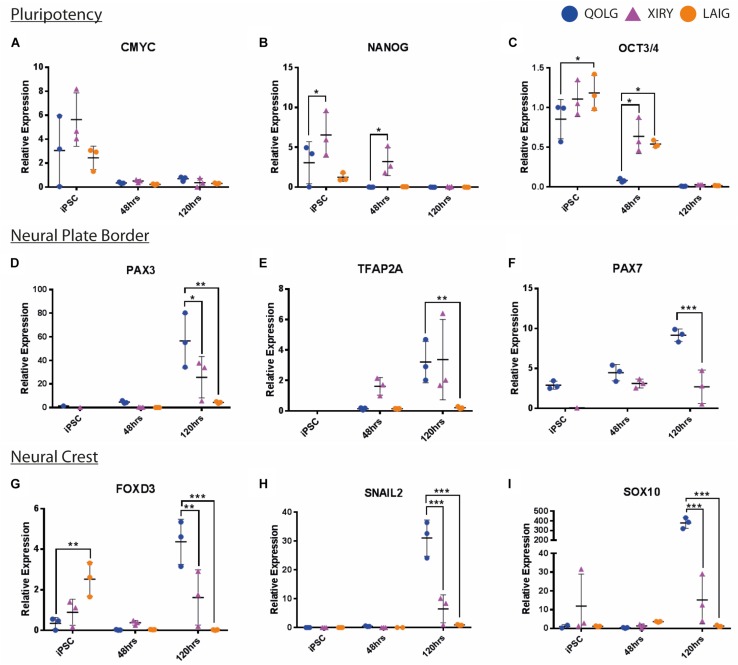
Dynamics of pluripotency, neural plate border and neural crest marker expression during neural crest induction. Neural crest induction was carried out on control QOLG (blue), mutant XIRY (purple), and LAIG (orange) hiPSC lines. Cells were harvested for RNA before plating (iPSC, time = 0 h), at 48 and 120 h of differentiation. RT-qPCR analysis was carried out for pluripotency markers [*CMYC* **(A)**, *NANOG* **(B),** and *OCT3/4* **(C)**], neural border identity [*PAX3* **(D)***, TFAP2A* **(E),** and *PAX7* **(F)**] and subsequently markers of neural crest identity [*FOXD3* **(G)***, SNAIL2* **(H)***, SOX10* **(I)]**. *P*-values were determined using unpaired Student’s *t*-tests (^*^P ≤ 0.05, ^∗∗^*P* ≤ 0.001, ^∗∗∗^*P* ≤ 0.0001).

To confirm the exit from pluripotency, we first analyzed the expression of *CMYC, NANOG*, and *OCT3/4*, which should become progressively reduced during the 120 h period. Indeed, these markers diminished as predicted in both QOLG and XIRY; however, it is worth noting that XIRY cultures exhibited a significantly higher level of all three pluripotency markers at the outset, including surprisingly higher levels of *NANOG* and *OCT3/4* at 48 h (Figures 6A–C). Interestingly, LAIG also expressed higher levels of *OCT3/4* at the iPSC period but not *NANOG* or *CMYC* (Figure 6C). By 120 h of culture, both lines had almost no expression of pluripotency markers. Nevertheless, the delayed loss of pluripotency markers could be indicative of a failure to respond to signals that induce the neural plate border.

We then examined expression of neural plate border markers, including *PAX3, PAX7, TFAP2A* (Figures 6D–F). As expected, the control line showed an increase in all three neural plate border markers over time, with steadily increasing levels at 48 and 120 h. In contrast, while there was some increase in all three markers at 48 h in XIRY compared to the start of the induction, neither *PAX3* nor *PAX7* reached the higher levels achieved in QOLG at 120 h (Figures 6D–F). LAIG also fails to upregulate neural plate border markers *PAX3* and *TFAP2A* (Figures 6D–E). For technical reasons, we were unable to assess *PAX7* expression in LAIG. Together, this demonstrates that induction of the neural plate border genes is not delayed, and is instead unable to reach the levels attained in controls. Altogether these results are strongly indicative of a failure to respond appropriately to inductive signals.

When we examined expression of the neural crest markers *FOXD3, SOX10*, and *SNAIL2* by qPCR, we found a profile similar to that of the neural border markers. While there was induction of all three genes during the differentiation protocol, expression levels in both mutant cell lines were significantly lower compared to control (Figures 6G–I). It is worth noting that prior to neural crest induction, LAIG expressed significantly higher levels of *FOXD3* (Figure 6G), which was lost by 48 h of induction. This raised the possibility that the mutant cell lines responded to the differentiation protocol via induction of a non-neural crest state. Note that we see some moderate induction of neural border and neural crest markers in XIRY.

**FIGURE 7:**
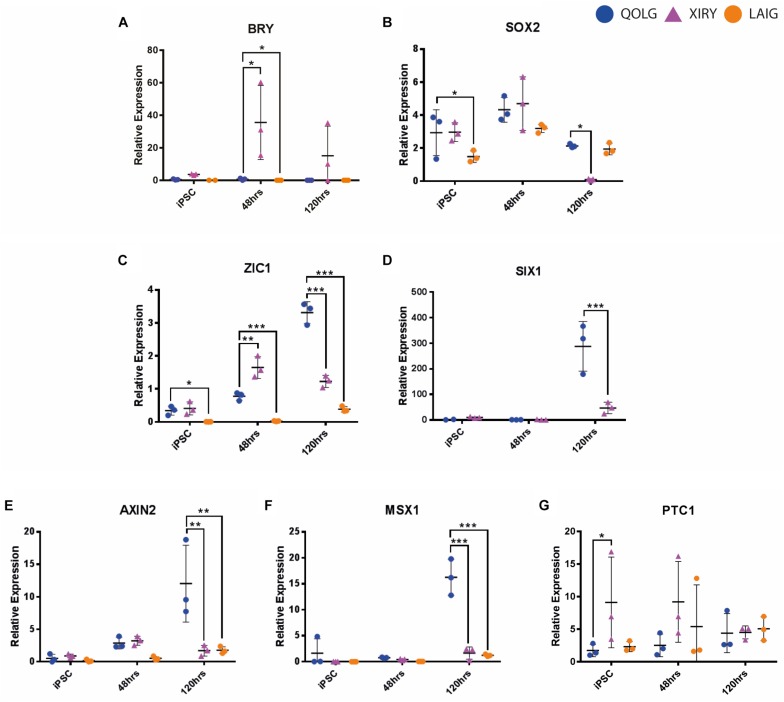
*BBS10* mutant line XIRY is skewed toward the mesodermal fate and has diminished WNT and BMP responsiveness. Neural crest induction was carried out on control QOLG (blue), mutant XIRY (purple), and LAIG (orange) hiPSC lines. Cells were harvested for RNA before plating (iPSC, time = 0 h), at 48 and 120 h of differentiation. **(A–D)** RT-qPCR analysis for a mesodermal marker, *BRY* **(A)**, a neural marker, *SOX2* **(B)**, neural border/pre-placodal marker, *ZIC1* **(C)** and pre-placodal marker *SIX1* **(D)**. Note the increased levels of *BRY* **(A)** and decreased levels of *ZIC1* **(C)** and *SIX1* **(D)** in XIRY. **(E–F)** RT-qPCR analysis for AXIN2 a transcriptional target of WNT signaling **(E)**, MSX1, a transcriptional target of BMP signaling **(F)** and PTC1, a transcriptional target of Hedgehog signaling **(G)**. **(E)** Note transient increase in AXIN2 expression in XIRY from 0 to 48 h, but a decrease by 120 h. **(F)** Note a failure to activate MSX1 expression in XIRY and LAIG compared to control QOLG. **(G)** Note variability of PTC1 expression in XIRY at 0 h and both mutants at 48 h. *P*-values were determined using unpaired Student’s *t*-tests (^*^P ≤ 0.05, ^∗∗^*P* ≤ 0.001, ^∗∗∗^*P* ≤ 0.0001).

Surprisingly, we found that XIRY expressed significantly higher levels of the mesodermal marker *Brachyury (BRY)* by 2 days of culture (Figure 7A). *SOX2* expression is an early marker of pluripotency (during the hiPSC stage) and then becomes a marker of neural tissues. Consistent with this, control QOLG cells express *SOX2* initially (0 h), followed by a peak at neural border stages (48 h) and a dip as neural crest induction progresses. In contrast, we found that XIRY cells had normal levels of *SOX2* initially, but this decreased significantly by day 5 of culture (Figure 7B). *SOX2* expression in the mutant LAIG was significantly lower during pluripotency stages, but responded normally to induction.

We next examined two markers of the pre-placodal ectoderm (PPE). Similar to the neural crest lineage, the pre-placodal ectoderm also relies on the appropriate induction of the neural border. *ZIC1* marks both the neural border and subsequently the PPE, and in controls, can be seen increasing over the course of the 5-day protocol (Figure 7C). To our surprise, *ZIC1* is significantly higher in XIRY mutant cells at 48 h but this trend is not continued, and levels of *ZIC1* in the mutant are significantly lower by 5 days of culture (Figure 7C). At all stages throughout induction, expression of *ZIC1* was significantly reduced in mutant LAIG. Sustained *ZIC1* expression is necessary for activation of pre-placodal ectoderm genes such as *SIX1* (Hong and Saint-Jeannet, 2007)*;* consistent with this, in XIRY, there is only a minimal increase of *SIX1* expression at 5 days compared to controls (Figure 7D). For technical reasons, we were unable to compare *SIX1* levels in LAIG.

Given the importance of BMP and WNT signals during specification of the neural plate border and subsequently in induction of the neural crest and pre-placodal lineages, we hypothesized that the mutant line had a dampened response to these key signals. To test this, we examined expression of the Wnt-responsive gene *Axin2* and the BMP-responsive gene *MSX1* (Figures 7E,F, respectively). Consistent with reports from Leung et al. (2016) we saw a graded increase in *AXIN2* expression in the control line QOLG during induction (Figure 7E). Both mutant lines showed drastically lower expression of *AXIN2* by 5 days of culture (Figure 7E). Similarly, the BMP-responsive gene *MSX1* failed to be up-regulated in the mutant lines (Figure 7F). Due to the crucial role for cilia in Hedgehog (Hh) signaling, we also examined the Hh target *Patched1 (PTC1*). There was generally no significant difference between control and mutant lines; however, mutants did show some variable increase early in induction (Figure 7G). This could indicate a lack of ciliary-mediated processing of the GLI repressors. Taken together, these data suggest that a failure in signal responsiveness in mutant cells underlies the observed neural crest induction phenotype.

## Discussion

Neurocristopathies, or diseases of the neural crest lineage, were first considered collectively by Bolande (1974). Since then, it has become clear that these disorders encompass a broad variety of diseases (Vega-Lopez et al., 2018). Due to the diversity of neural crest derivatives, mutations affecting this lineage lead to many pleiotropic phenotypes. Furthermore, the assimilation of the neural crest lineage into multiple organ systems makes it particularly difficult to assess function of this lineage *in vitro*. Therefore, recent advances in human-derived cellular approaches offer new tools for the study of complex human conditions. To be able to best use these patient-derived cells, there is an acute need to establish streamlined, robust and reproducible assays to test each step of neural crest formation. In this work, we used the short neural crest induction protocol developed by Leung et al. (2016) to determine key requirements for a disease gene in neural crest stem cell pathology.

The long-term goal of a project such as this is the ability to model “disease in a dish,” using the patient’s own cells to understand the underlying cellular and molecular causes of pathology or using the patient’s cells to design new therapeutics. This approach complements existing animal models, which are very powerful for *in vivo* studies, but often do not entirely recapitulate the human condition. For example, genetic knock-outs of crucial genes frequently lead to embryonic lethality. However, it is clear from disorders such as BBS, that the human disease variants are often of unknown function and difficult to study without a clear cellular or molecular assay. Furthermore, genes such as *BBS10* lead to a broad range of phenotypes in patients, including different manifestations from sibling to sibling (carrying the same variant). This variability also complicates our understanding of gene-function interactions.

To truly link gene identity to gene function using animal models, it would be necessary to generate “humanized” models: e.g., knock-ins of human disease gene variants. This is clearly not feasible in most cases, and, may not truly reflect the human condition due to genetic background. While use of hiPSCs to complement animal models has become more common in recent years [e.g., with *CHD7* patients (Okuno et al., 2017)], there has not yet been an effort to systematically screen candidate neurocristopathy patients. Although this study is only focused on two *BBS10* mutant lines, our work clearly illustrates the power of defined protocols to link gene variation to gene function.

Bardet–Biedl Syndrome *10* is a ciliopathy gene, and is one of the most common genes mutated in BBS. Despite our knowledge of multiple BBS human disease variants, it has been difficult to understand the direct causative mechanisms underlying the human condition. An established differentiation protocol such as the neural crest induction assay is an important tool that will allow us to dissect out the gene variants that are pathogenic. As the CRISPR/Cas9 approaches become more efficient, we should be able to selectively repair the BBS variants in these lines, or to generate “disease” variants in control lines. Alternatively, over-expression analysis with a wildtype construct might be sufficient to rescue this neural crest induction phenotype, while overexpression with different variants would allow us to test specific mutations for gain- or loss-of-function during neural crest induction. This will allow us to directly compare the effect of different variants on the same genetic background in order to help us begin to understand the pleiotropic effects seen in humans.

Applications of an assay such as this one are also broad. We can use this protocol to understand both the cellular and molecular profiles of patient specific cells. For example, when Leung et al. (2016) initially developed the short-term protocol, they tested the requirements of fibroblast growth factor (FGF) and bone morphogenetic protein (BMP) signaling during the induction phase. They noted that excessive levels of FGF led to increased mesodermal specification, while BMP4 supplementation led to increased non-neural ectoderm. In contrast, BMP inhibition reduced the levels of SOX10 positive neural crest cells. As *BBS10* is a key ciliopathy gene, and the cilium can serve as a node for signal transduction, it was conceivable that the phenotypes seen in our *BBS10* hiPSCs were due to an inability to respond to the appropriate inductive cues. As the main driver of the neural crest induction protocol in this assay is Wnt activation via GSK3 inhibition, our key finding here suggests that the ciliopathic hiPSCs have a diminished ability to respond to the GSK3 inhibitor CHIR99021, which is included throughout the induction protocol. Indeed, the XIRY line initially responded to CHIR99021 by upregulating the Wnt target gene *AXIN2* modestly; however, further treatment did not recapitulate the upregulation seen in QOLG at 120 h (Figure 7E). XIRY also fails to activate expression of the BMP target gene *MSX1*. At this point, it is unclear whether XIRY is unable to respond, or whether there is another overriding signal. In the long-term, a molecular analysis such as RNAseq would be a more thorough way to compare responsiveness of wildtype and mutant cells. Nevertheless, our findings provide an intriguing starting point for understanding the inductive cues necessary for neural crest development.

To our knowledge, our study is the first exploration of the requirements for ciliopathy genes in human neural crest development. Our findings correlate with previous observations regarding BBS function in zebrafish models of neural crest development (Tobin et al., 2008), and suggests a potential role for cilia genes during establishment of the neural plate border and induction of the neural crest lineage. Having established this pipeline, these assays can be optimized to probe some key characteristics of neural crest cells. For example, two of the defining features of neural crest cells are the prodigious migratory capacity and the multipotency. Endpoint and live high-content imaging assays can be developed using these read outs. As we can already see from our studies, *BBS10* mutant cells have very different morphology, protrusive activity and migratory capacity (data not shown). In fact, some ciliopathies, including BBS, have been associated with Hirschsprung’s disease, which is characterized by a failure of migration in neural crest-derived enteric neurons (de Pontual et al., 2009). We expect to be able to synchronize our cell populations to readily and directly compare the cellular behaviors (as in Wiseman et al., 2019). With regards to the differentiation capacity of neural crest, it should be immediately possible to adapt these cultures toward the differentiation of the diverse cell types produced by the neural crest, including bone, cartilage, adipocytes, neurons, pigment and other cell types.

Overall, the use of patient derived hiPSCs for the modeling of neurocristopathy phenotypes can elucidate the requirement of specific genes at crucial points of neural crest development. This human genetic background specific approach in combination with animal models will lead to a greater understanding of mammalian neural crest development and disease.

## Author Contributions

WB and KL designed the study, produced, collected and interpreted data, and wrote the manuscript. JG and J-LH performed the experiments and collected the data. AG provided help with experimental design and interpretation of findings. HipSci Consortium, DD, and PB provided the crucial reagents and expertise. All authors contributed to and approved the manuscript.

## Conflict of Interest Statement

The authors declare that the research was conducted in the absence of any commercial or financial relationships that could be construed as a potential conflict of interest.

## Footnotes

1 http://www.hipsci.org
